# Monic Chebyshev pseudospectral differentiation matrices for higher-order IVPs and BVPs: applications to certain types of real-life problems

**DOI:** 10.1007/s40314-022-01940-0

**Published:** 2022-07-20

**Authors:** M. Abdelhakem, A. Ahmed, D. Baleanu, M. El-kady

**Affiliations:** 1 Department of Mathematics, Faculty of Science, Helwan University, Cairo, Egypt; 2 Basic Science Department, School of Engineering, Canadian International College (CIC), New Cairo, Egypt; 3 Department of Mathematics, Cankaya University, Ankara, Turkey; 4 Institute of Space Sciences, Magurele-Bucharest, Romania; 5 Lebanese American University, 11022801 Beirut, Lebanon

**Keywords:** Monic Chebyshev polynomials, Pseudospectral differentiation matrices, Convergence and error analysis, Higher-order IVPs and BVPs, MHD, COVID-19, Monic Chebyshev polynomials, Pseudospectral differentiation matrices, Convergence and error analysis, Higher-order IVPs and BVPs, MHD, COVID-19, 65L05, 65L10, 65L20, 65L70, 76M22, 33C47

## Abstract

We introduce new differentiation matrices based on the pseudospectral collocation method. Monic Chebyshev polynomials (MCPs) were used as trial functions in differentiation matrices (D-matrices). Those matrices have been used to approximate the solutions of higher-order ordinary differential equations (H-ODEs). Two techniques will be used in this work. The first technique is a direct approximation of the H-ODE. While the second technique depends on transforming the H-ODE into a system of lower order ODEs. We discuss the error analysis of these D-matrices in-depth. Also, the approximation and truncation error convergence have been presented to improve the error analysis. Some numerical test functions and examples are illustrated to show the constructed D-matrices’ efficiency and accuracy.

## Introduction

Initial value problems (IVPs) and boundary value problems (BVPs) demonstrate many branches of science. Some of them are in the field of engineering, technology, optimization theory, and classical mechanics (Ribeiro and de-Sousa 2018). Moreover, there is astonishing growth in interest in problems associated with systems of linear and nonlinear ODEs. Models for COVID-19 built using these systems (Farman et al. 2021; Rong et al. 2020; Moore and Okyere 2022). Also, many applications are described by higher-order BVPs as in Magnetohydrodynamic flow (Karkeraa et al. 2020) and transverse vibration of a uniform beam (Khalil et al. 2012; El-Kady et al. 2014). Other advanced models are formed by a system of nonlinear higher-order BVPs as in Eid et al. (2020), Alsaedi et al. (2020), Danish et al. (2021) and Abo-Eldahab et al. (2021) for the nanofluid flow and in Subhashini et al. (2020) for mixed convection flow. As a generalization, the application extended to the fractional calculus as represented in Boukhouima et al. (2020), Abdelhakem et al. (2019a, 2021a).

Since the BVPs have wide applications in scientific research, hence it has been important to find numerical methods for solving these problems. Those problems haven’t been solved analytically (Youssri et al. 2021; Nayak and Khan 2020; Reddy 2016).

Spectral methods solved ODEs by expressing these equations in terms of a series of known functions (Abdelhakem et al. 2019b). The basic concept of any spectral method is to use trial functions, called basis or expansion approximating functions. Very smooth, global, and orthogonal are considered to be the vital properties of these polynomials. Spectral methods involved three types, namely the Galerkin (Elahi et al. 2018; Kasi Viswanadham and Kiranmayi Ch 2017), Tau (Abdelhakem and Youssri 2021), and Collocation methods (Sohaib et al. 2018; Abdelhakem et al. 2021b). In the spectral collocation method, we enforce the numerical solution to almost validate the problem as closely as possible. Thus, the residuals may be allowed to be zero at specific collocation nodes.

Another technique came from the collocation method, which is the pseudospectral method (Abdelhakem et al. 2022). Many authors use different types of extreme points and weights in pseudospectral methods. The main points and weights are Gauss Quadrature (GQ), Gauss–Radau (GR), and Gauss–Lobatto-quadrature (GLQ) (Shen et al. 2011). However, others may use the equally spaced (El-Kady et al. 2014; Akram et al. 2017; Hassan 2004). Higher-order BVPs have been solved by pseudospectral D-matrices using different polynomials. The efficiency and accuracy of these methods have been proved (Abdelhakem et al. 2020; Elbarbary and El-Sayed Salah 2005).

Herein, the MCPs have been used as trial functions. These will be used via pseudospectral to construct pseudospectral D-matrices. The advantage of the pseudospectral D-matrices is that it is not used to solve the differential equation as the spectral operational matrices. They can be used as differentiation tools to differentiate several functions with high accuracy. On the other hand, the leading coefficient of the monic polynomials is one. Hence, the presented basis functions, MCPs, are generated by dividing the Chebyshev polynomials by $2^{1-n}$, where $n>0$ is the polynomials degree. Due to this definition, the spectral expansion will converge rapidly. This will be discussed later in a separate section, and its effect will be reported in the numerical examples.

The outline of this paper is: in Sect. 2, preliminaries and concepts that are needed through this article are introduced. We include a brief summary of Chebyshev polynomials (CPs) and MCPs. Also, some useful definitions and relations for the MCPs are mentioned. In Sect. 3, we investigate some properties, concepts, and relations of MCPs. Moreover, the discrete weights and the zeros of MCPs are constructed. Orthogonal relations of MC function are generated. In Sect. 4, new pseudospectral D-matrices of MCPs are constructed. The error analysis to estimate the error bounds of the MC approximations has been derived in Sect. 5. In Sect. 6, we applied the MC approximation for some test functions and compared the obtained results with others. The proposed method and how to use the MC D-matrices were introduced in Sect. 7. Through Sect. 8, two techniques are applied to solve H-ODEs. The first technique, solves the H-ODEs directly using the MC D-matrices. The second technique transforms the H-ODE to a system of lower order ODEs. The obtained results are compared with other methods and the bvp5c MATLAB (if possible) function to show the accuracy and efficiency of MC D-matrices.

## Preliminaries and notations

In this section, we shall introduce CPs and MCPs. Then, we give a brief summary of the pseudospectral method.

### Chebyshev and monic Chebyshev polynomials

The CPs of degree *n* ($T_n(x), n = 0,1,2, \dots ; x\in [-1,1])$ are the solutions of the Chebyshev differential equation (Mason and Handscomb 2002):

1 $$\begin{aligned} (1-x^2 )y^{''}(x)-xy^{'}(x)+n^2y(x)=0\, , \end{aligned}$$

where,

2 $$\begin{aligned} T_0 (x)=1, \quad T_1 (x)=x, \quad T_2 (x)=2x^2-1. \end{aligned}$$

Also, CPs ($T_n(x);n>1$) can be obtained through the recursive formula:

3 $$\begin{aligned} T_{n+1}(x)=2xT_n (x)-T_{n-1}(x);\quad n=1, 2, 3, \dots \, , \end{aligned}$$

with the initials $T_0 (x)=1$ and $T_1 (x)=x$.

The definition of CPs yields the bounds:

4 $$\begin{aligned} |T_n(x)|\le 1,\quad |T_n^{'} (x)|\le n^2\, , \end{aligned}$$

with the boundary values:

5 $$\begin{aligned} T_n(\pm 1)=(\pm 1)^n,\quad T_n^{'}(\pm 1)=(\pm 1)^n n^2. \end{aligned}$$

Derivatives of the recursive relation of CPs are

6 $$\begin{aligned} T_{n+1}^{'}(x)=2T_n(x)+2xT_n^{'}(x)-T_{n-1}^{'}(x); \quad n=2, 3, \dots . \end{aligned}$$

Another definition of CPs of the first kind is defined through the identity:

7 $$\begin{aligned} T_n (x)=\cos (n\theta )\, , \end{aligned}$$

where $$\theta =\cos ^{-1}(x)$$ and $$x\in [-1,1]$$.

Let $``Q_{n}(x);\, n=0,1,2,....\, ;\, x\in [-1,1]''$ be the MCPs of the first kind (El-Kady and Moussa 2013). The unique system of MCPs $\{Q_n\}$ is defined by:

8 $$\begin{aligned} Q_{n}(x)=\left\{ \begin{array}{ccc} 1&{},&{} n=0,\\ 2^{1-n}T_{n}(x)&{}, &{} n\ge 1. \end{array} \right. \end{aligned}$$

Using relation (8), we have:

9 $$\begin{aligned} Q_{1}(x)=x,\quad Q_{2}(x)=x^{2}-\frac{1}{2} \end{aligned}$$

The recursive formula of MCPs is:

10 $$\begin{aligned} Q_{n}(x)=xQ_{n-1}(x)-\frac{1}{4}Q_{n-2}(x);\quad n\ge 3 \end{aligned}$$

The the recursive relation for MCPs in terms of its derivatives is Abdelhakem et al. 2019b:

11 $$\begin{aligned} Q_{n}(x)=\frac{1}{n+1} Q_{n+1}^{'}(x)-\frac{1}{4(n-1)}Q_{n-1}^{'}(x); n\ge 2. \end{aligned}$$

The MCPs constitute an orthogonal basis w.r.t. $w(x)=1/\sqrt{1-x^2}$ (the same weight of CPs):

12 $$\begin{aligned} \left( Q_{i},Q_{j}\right) =\int _{-1}^{1}Q_{i}(x)Q_{j}(x)w(x)\mathrm{{d}}x=\left\{ \begin{array}{ccc} 0&{}, &{} i\ne j\, ,\\ 2^{1-2i}\pi &{}, &{} i=j\ne 0\, , \\ \pi &{}, &{} i=j=0\, . \end{array} \right. \end{aligned}$$

### Gauss–Lobatto quadrature

Throughout, this paper we shall use Gauss–Lobatto quadrature (GLQ) as the collocation points . Let $\{q_n(x)\}_{n=0}^{\infty }$, defined on the interval [*u*, *v*], be orthogonal polynomials w.r.t the weight function *w*(*x*). Then (Shen et al. 2011):

13 $$\begin{aligned} U_{N-1}(x)=\frac{q_{N+1}(x)+\alpha q_{N}(x)+\beta q_{N-1}(x)}{(x-u)(v-x)}\, , \end{aligned}$$

where $\alpha , \beta$ are given by solving the equation:

14 $$\begin{aligned} q_{N+1}(x)+\alpha q_{N}(x)+\beta q_{N-1}(x)=0 \, ;\quad x=u,v. \end{aligned}$$

#### Definition 2.1

(Shen et al. 2011) The inner product of the orthogonal polynomials $\{q_n(x)\}_{n=0}^{\infty }$ w.r.t the weight function *w*(*x*) over the interval [*u*, *v*], denoted by $\left( q_n,q_n \right) _{w}=\left| \left| q_n\right| \right| _w^2$, is defined by:

15 $$\begin{aligned} (q_n,q_n)_{w}=\left| \left| q_n\right| \right| _w^2=\int _{u}^{v}q_n^2(x)w(x)\mathrm{{d}}x \, . \end{aligned}$$

#### Definition 2.2

(Shen et al. 2011) The GLQ points of the orthogonal polynomials $q_n(x)$ are the zeros of function (13) and the ends points *u*, *v*.

#### Lemma 1

(Shen et al. 2011) Let $\{x_s\}_0^N$ be the GLQ points of the orthogonal polynomials $q_n(x)$. Then $\{x_s\}_0^N$ are the zeros of the equation:

16 $$\begin{aligned} (x-u)(v-x)\, q'_{N}(x)=0\, . \end{aligned}$$

#### Theorem 2

(Shen et al. 2011) Let $\{x_s\}_0^N$ be the GLQ points of the orthogonal polynomials $q_n(x)$. Then, there is a unique set of quadrature weight (QW) $\{w_s\}_0^N$ given by:

17 $$\begin{aligned} w_0=\frac{1}{(v-u)U_{N-1}(u)}\int _{u}^{v}(1-x)U_{N-1}(x)w(x)\mathrm{{d}}x \, , \end{aligned}$$

18 $$\begin{aligned} w_s=\frac{1}{(x_s-u)(v-x_s)}\frac{k_{N+1}}{k_N}\frac{\left\| U_{N-2}\right\| _{\hat{w}}}{U_{N-2}(x_s)U^{'}_{N-1}(x_s)} \, ,\quad 0<s<N\, , \end{aligned}$$

19 $$\begin{aligned} w_N=\frac{1}{(v-u)U_{N-1}(v)}\int _{u}^{v}(x-u)U_{N-1}(x)w(x)\mathrm{{d}}x \, , \end{aligned}$$

such that

20 $$\begin{aligned} \int _{u}^{v}q(x)w(x)\mathrm{{d}}x=\sum _{j=0}^{N}q(x_j)w_j;\quad \forall q\in P_{2N-1} \, , \end{aligned}$$

where $k_N$ is the leading coefficient of the polynomial $q_N(x)$   ,

21 $$\begin{aligned} \hat{w}(x)=(x-u)(v-x)w(x) \, , \end{aligned}$$

and $\left\| U_{N-2}\right\| _{\hat{w}}^2$ is the inner product of $U_{N-2}$ with respect to $\hat{w}$.

#### Definition 2.3

(Shen et al. 2011) The discrete inner product of the orthogonal polynomials $\{q_n(x)\}_{n=0}^{\infty }$ with respect to the weight function $w_j$, denoted by $\left\langle q_n,q_n \right\rangle _{N,w}=\left\| q_n\right\| _{N,w}^2$ is defined by:

22 $$\begin{aligned} \left\langle q_n,q_n \right\rangle _{N,w}=\left\| q_n\right\| _{N,w}^2=\sum _{j=0}^{N}q_n^2(x_j)w_j. \end{aligned}$$

By using Eqs. (16), (17), (18), and (19), we get the Chebyshev GLQ of the CPs (C-GLQ) point as:

23 $$\begin{aligned} x_s=\cos \frac{\pi s}{N};\quad 0\le s\le N\,, \end{aligned}$$

and the Chebyshev QW (C-QW):

24 $$\begin{aligned} w_s=\frac{\pi \theta _{s}}{N};\quad 0\le s\le N\,, \end{aligned}$$

where $\theta _0=\theta _N=1/2$ and $\theta _s=1$; $0<s<N$. By using above definitions and properties, we have the following theorem.

#### Theorem 3

(Shen et al. 2011) The discrete inner product of CPs is defined as:

25 $$\begin{aligned} \left\langle T_n,T_m \right\rangle _{n,w}={\left\{ \begin{array}{ll} 0, &{} \text {if }n\ne m \, ,\\ \pi , &{}\text {if }n=m=0 \,\text {and } n=m=N \, ,\\ \frac{\pi }{2}, &{}\text {if }0<m, n<N\, \text {and } n=m \, . \end{array}\right. } \end{aligned}$$

### Pseudospectral method

The pseudospectral method is a technique in which the unknown function *f*(*x*) of the ODEs is still approximated as in a spectral method:

26 $$\begin{aligned} f(x)=\sum _{k=0}^N a_k q_k(x) \, . \end{aligned}$$

Use the discrete inner product with $\{x_{j},w_j\}_{j=0}^{N}$ as associated GLQ points with the QW to get:

27 $$\begin{aligned} f(x)=\sum _{j=0}^{N}\sum _{n=0}^{N}\frac{w_j}{\Vert q_n\Vert _{N,w}^2}q_n (x_j)f(x_j)q_n(x) \, . \end{aligned}$$

This approximation is actually represented not by its coefficients but by the values of the unknown function $f(x_j )$ at $(N+1)$ GLQ points $x_j$ ,$j=0, 1, 2,\dots ,N$ (Shen et al. 2011).

## On monic Chebyshev polynomials

“If a single flap of a butterfly’s wings can be instrumental in generating a tornado” (Lorenz 1993)—Professor Edward Lorenz. As mentioned in the above sections, the only difference between MCPs and CPs is the leading coefficient (The single flap). But in the results, we recognized a huge difference (The tornado). The effect of this difference will be shown in the rapid rate of convergence (Sect. 5.3).

This section aims to present the properties of MCPs. Some theorems for MCPs will be presented, such as QW (MC-QW), the constants of finite expansion for *f*(*x*), the zeros (MC-GLQ) of MCPs, and the discrete inner product.

### Monic Chebyshev Gauss–Lobatto quadrature weight

In this section, the MC-QW will be deductive. The importance of that weight comes from them that it’s needed to discuss the discrete orthogonal relation of MCPs.

#### Lemma 4

According to Eq. (13):

28 $$\begin{aligned} U_{N-1}(x)=\frac{Q_{N+1}(x)-\frac{1}{4}Q_{N-1}(x)}{1-x^{2}}. \end{aligned}$$

#### Proof

Use Eq. (14) to determine the values of $\alpha ,\beta$. So, the equation takes the form:

29 $$\begin{aligned} Q_{N+1}(x)+\alpha Q_{N}(x)+\beta Q_{N-1}(x)=0\, , x=-1,1. \end{aligned}$$

According to Eqs. (8) and (5) we get

30 $$\begin{aligned} 2^{-N}+2^{1-N}\alpha +2^{2-N}\beta =0\,. \end{aligned}$$

and

31 $$\begin{aligned} 2^{-N}(-1)^{N+1}+2^{1-N}(-1)^{N}\alpha +2^{2-N}(-1)^{N-1}\beta =0\,. \end{aligned}$$

Solving Eqs. (30) and (31) to get $\alpha =0$ and $\beta ={-1}/{4}$. Finally, using the values of $\alpha$ and $\beta$ with Eq. (13) to complete the proof.

Note that, the above function, (28), is different from its form in CPs.

#### Definition 3.1

The MC-GLQ points of the orthogonal polynomials $Q_n(x)$, $\{{x_s}\}_{s=0}^N$, are the zeros of function (28).

#### Lemma 5

Let $\{{x_{s}}\}_{0}^{N}$ be the MC-GLQ points of the orthogonal polynomials $Q_{n}(x)$. Then:

32 $$\begin{aligned} \{{x_{s}}\}_{0}^{N}=\left\{ {cos\frac{\pi s}{N} }\right\} _{0}^{N}. \end{aligned}$$

#### Proof

Straightforward by equating Eq. (28) by zero.

We noted that the zeros of MCPs are the same as CPs’ zeros.

#### Lemma 6

Let $\{{x_{s}}\}_{0}^{N}$ be MC-GLQ points. Then, MC-QWs are:

33 $$\begin{aligned} w_s^*= \frac{\theta _s\pi }{N} \, , \end{aligned}$$

where

34 $$\begin{aligned} \theta _{s}=\left\{ \begin{array}{cc} \frac{1}{2}, \,\,\,&{} s=0,N \, , \\ 1, \,\,\,&{} 0<s<N \, . \end{array} \right. \end{aligned}$$

#### Proof

To find the $w^{*}_0$: from Lemma (4), use $x=-1$ to get:

35 $$\begin{aligned} U_{N-1}(-1)=\lim _{x\rightarrow -1}\frac{Q_{N+1}(x)-\frac{1}{4}Q_{N-1}(x)}{1-x^{2}}\, . \end{aligned}$$

By using L’Hopital’s rule and the boundary properties of MCPs:

36 $$\begin{aligned} U_{N-1}(-1)=\frac{2N}{2^N}(-1)^{N}\, . \end{aligned}$$

Substituting from Eqs. (28) and (36) into Eq. (17):

37 $$\begin{aligned} w_0^{*}&=\frac{1}{(v-u)U_{N-1}(u)}\int _{u}^{v}(v-x)U_{N-1}(x)w(x)\mathrm{{d}}x\nonumber \\&=\frac{2^{N}}{4N(-1)^{N}} \int _{-1}^{1} (1-x) \frac{Q_{N+1}(x)-\frac{1}{4}Q_{N-1}(x)}{1-x^{2}}\frac{1}{\sqrt{1-x^{2}}} \mathrm{{d}}x\,. \end{aligned}$$

Use $$x= \cos \theta $$, then:

$$\begin{aligned} w_0^{*}&=\frac{-2^{N}}{4N(-1)^{N}} \int _{\pi }^{0}\left[ \frac{2^{-N} \cos \left( (N+1) \theta \right) }{1+ \cos \theta }-\frac{2^{-N-2}\cos \left( (N-1) \theta \right) }{4(1+ \cos \theta )}\right] \mathrm{{d}} \theta \\&=\frac{-1}{4N(-1)^{N}} \int _{0}^{\pi }\frac{\cos (N \theta ) \sin \theta }{\cos \theta +1}=\frac{-1}{4N(-1)^{N}}(-\pi \cos (N \pi )) \mathrm{{d}}\theta . \end{aligned}$$

Thus:

38 $$\begin{aligned} w_0^*=\frac{\pi }{2N}=\frac{\theta _0\pi }{N} \, . \end{aligned}$$

Similarly,

39 $$\begin{aligned} w^{*}_{N}=\frac{\pi }{2N}=\frac{\theta _N\pi }{N} \, . \end{aligned}$$

For, $$w_{s}^{*}$$; $$0<s<N$$:

The leading coefficients of MCPs are always equal to 1. So, $k_{N+1}=k_{N}=1$. Since the zeros of MC-GLQ are $x_{s}=\cos \left( s\pi /N\right)$. Then, $(x_{s}+1)(1-x_{s})=1-x_{s}^{2}=1-\cos ^{2}(s\pi /N)$. Thus, from Lemma (4), replace $N-1$ by $N-2$:

40 $$\begin{aligned} U_{N-2}(x)&=\frac{Q_{N}(x)-\frac{1}{4}Q_{N-2}(x)}{1-x^{2}}=\frac{2^{1-N}\cos (N\theta )-\frac{1}{4} 2^{3-N}\cos ((N-2)\theta )}{1-\cos ^{2}\theta }\nonumber \\&=\frac{2^{1-N}(\cos (N\theta )-\cos (N\theta ) \cos (2\theta )-\sin (N\theta )\sin (2\theta ))}{1-\cos ^{2}\theta }\, . \end{aligned}$$

Since $x_s$ is equivalent to $\theta _s=\frac{s \pi }{N}$, thus:

41 $$\begin{aligned} U_{N-2}(x_s)&=\frac{2^{1-N}(\cos (N\frac{s \pi }{N})-\cos (N\frac{s \pi }{N}) \cos (2\frac{s \pi }{N})-\sin (N\frac{s \pi }{N})\sin (2\frac{s \pi }{N}))}{1-\cos ^{2}\frac{s \pi }{N}}\nonumber \\&=\frac{2^{1-N}(-1)^s(1-\cos (2\frac{s \pi }{N}))}{\sin ^{2}\frac{s \pi }{N}}\nonumber \\&=2^{2-N}(-1)^s\, . \end{aligned}$$

Also,

42 $$\begin{aligned} ||U_{N-2}(x)||^{2}_{\hat{w}}&=\int _{-1}^{1}U_{N-2}^{2}(x)\hat{w}(x) \mathrm{{d}}x=\int _{-1}^{1}U_{N-2}^2 w(x)(1-x^2) \mathrm{{d}}x \nonumber \\&=-\int _{0}^{\pi }\frac{(2^{1-N}\cos (N\theta )-\frac{1}{4}2^{3-N}\cos ((N-2)\theta ))^{2}}{\sin ^{2}\theta } \mathrm{{d}}\theta \nonumber \\&=-2^{2-2N}\int _{0}^{\pi }\frac{(2\cos (N\theta )\sin ^{2}\theta -2\sin (N\theta )\sin \theta \cos \theta )^{2}}{\sin ^{2}\theta }\mathrm{{d}} \theta \nonumber \\&=-4\times 2^{2-2N}\int _{0}^{\pi }\left[ \sin ((N-1)\theta )\right] ^{2}\mathrm{{d}}\theta \nonumber \\&=-2^{3-2N}\pi \, . \end{aligned}$$

Furthermore, $U_{N-1}^{'}(x)$ is needed. So by replacing *x* with $\cos \theta$ in Eq. (28):

43 $$\begin{aligned} U_{N-1}(x)&=-2^{1-N}\frac{\sin (N\theta )}{\sin \theta } \, . \end{aligned}$$

Then,

44 $$\begin{aligned} U_{N-1}^{'}(x)=2^{1-N}\frac{N\sin \theta \cos (N\theta )-\sin (N\theta )\cos \theta }{\sin ^{3}\theta } \, . \end{aligned}$$

Thus,

45 $$\begin{aligned} U_{N-1}^{'}(x_s)&=-2^{1-N}\frac{(-1)^{s}}{\sin ^{2}(\frac{s \pi }{N})} \, . \end{aligned}$$

Substitute from Eqs. (42), (41), and (45) into Eq. (18) to get $w_{s}^{*}$.

### Orthogonality of monic Chebyshev polynomials

The importance of this relation comes from its use to set up the D-matrices.

#### Theorem 7

The discrete inner product of MCPs is:

46 $$\begin{aligned} \left\langle Q_n,Q_m \right\rangle _{N,w^*}=\left\{ \begin{array}{ccl} 0&{}, &{} \text {if }n\ne m \, ,\\ \pi &{}, &{}\text {if }n=m=0 \, , \\ 2^{2-2N}\pi &{}, &{}\text {if }n=m=N \, , \\ 2^{1-2n}\pi &{}, &{}\text {if }0<m, n<N \text {and } n=m \, . \end{array} \right. \end{aligned}$$

#### Proof

Straightforward using Theorem 3.

## Monic Chebyshev differentiation matrices

In this section, some important theorems and lemmas have been presented. These theorems and lemmas are needed to set up the MC D-matrices.

### Lemma 8

Let *f*(*x*) be a continuous function that can be approximated by the MC approximation over N+1 MC-GLQ points as:

47 $$\begin{aligned} f(x)=\sum _{n=0}^{N}a_{n}Q_{n}(x). \end{aligned}$$

Then,

48 $$\begin{aligned} a_{n}=\frac{2^{2n-1}}{N}\sum _{j=0}^{N} c_{n}f(x_{j})Q_{n}(x_{j})\theta _{j} \, , \end{aligned}$$

such that,

49 $$\begin{aligned} \theta _{j}=\left\{ \begin{array}{cc} \frac{1}{2}, &{} j=0,N \, ,\\ 1, &{} 0<j<N \, , \end{array} \right. and c_{n}=\left\{ \begin{array}{ccc} 2, &{} n=0 \, , \\ 1, &{} 0<n<N\, , \\ \frac{1}{2}, &{} n=N \, . \end{array} \right. \end{aligned}$$

### Proof

Since, $f(x)=\sum \nolimits _{n=0}^{N}a_{n}Q_{n}(x)$. Then, from Definition (2.3) $a_n=\frac{1}{\left\langle Q_n,Q_n\right\rangle _{N,w} }\sum _{j=0}^{N} f(x_{j})Q_{n}(x_{j})w^*_j$. By using MC-QW (Eq. (33)):

50 $$\begin{aligned} a_n=\frac{1}{\left\langle Q_n,Q_n\right\rangle _{N,w} }\sum _{j=0}^{N} f(x_{j})Q_{n}(x_{j})\frac{\theta _j\pi }{N} \, . \end{aligned}$$

Now, according to Theorem (7):

At $$n=0$$:

51 $$\begin{aligned} a_n=\frac{1}{\pi }\sum _{j=0}^{N} f(x_{j})Q_{n}(x_{j})\frac{\theta _j\pi }{N}=\frac{1}{N}\sum _{j=0}^{N} f(x_{j})Q_{n}(x_{j})\theta _j , \end{aligned}$$

and for $$0<n<N$$:

52 $$\begin{aligned} a_n=\frac{1}{2^{1-2n}\pi }\sum _{j=0}^{N} f(x_{j})Q_{n}(x_{j})\frac{\theta _j\pi }{N}=\frac{2^{2n-1}}{N}\sum _{j=0}^{N}f(x_j)Q_n(x_j)\theta _{j} \, . \end{aligned}$$

Finally, at $$n=N$$:

53 $$\begin{aligned} a_n=\frac{1}{2^{2-2N}\pi }\sum _{j=0}^{N} f(x_{j})Q_{n}(x_{j})\frac{\theta _j\pi }{N} =\frac{2^{2N-2}}{N}\sum _{j=0}^{N} f(x_{j})Q_{n}(x_{j})\theta _j \, . \end{aligned}$$

Hence, the lemma is proved.

It is clear that, there is a slight difference between the constants, $c_n: n=0,1, \dots , N$, and those in CPs (Elbarbary and El-Sayed Salah 2005).

Let *f*(*x*) be $r+1$, r is a positive integer, differentiable function on the interval $[-1,1]$. Since, $f^{(r)}(x)$ and $f^{(r+1)}(x)$ are two continuous functions on the interval $[-1,1]$. Then, form Eq. (47):

54 $$\begin{aligned} f^{(r)}(x)=\sum _{n=0}^{N}a_{n}^{(r)}Q_{n}(x), \end{aligned}$$

and

55 $$\begin{aligned} f^{(r+1)}(x)&=\sum _{n=0}^{N} a_{n}^{(r+1)}Q_{n}(x)=a_{0}^{(r+1)}+a_{1}^{(r+1)}x+\sum _{n=2}^{N}a_{n}^{(r+1)}Q_{n}(x) \nonumber \\&=a_{0}^{(r+1)}+a_{1}^{(r+1)} x+\sum _{n=2}^{N}a_{n}^{(r+1)}\left( \frac{1}{n+1}Q_{n+1}^{'}(x)-\frac{1}{4(n-1)}Q_{n-1}^{'}(x)\right) \nonumber \\&=\sum _{n=1}^{N}Q_{n}^{'}(x)\left( \frac{1}{n}a_{n-1}^{(r+1)}-\frac{1}{4n}a_{n+1}^{(r+1)}\right) \, . \end{aligned}$$

By differentiating Eq. (54) w.r.t. *x*:

56 $$\begin{aligned} f^{(r+1)}(x)=\sum _{n=1}^{N}a_{n}^{(r)}Q_{n}^{'}(x) \, . \end{aligned}$$

By equating the coefficients of $Q_{n}^{'}(x)$ of Eq. (55) with Eq. (56), we have:

57 $$\begin{aligned} a_{n}^{(r)}=\frac{1}{n}a_{n-1}^{(r+1)}-\frac{1}{4n}a_{n+1}^{(r+1)} \, . \end{aligned}$$

This difference equation can be solved to give:

58 $$\begin{aligned} a_{n}^{(r)}=\sum _{i=1}^{\infty }2^{2-2i}(n+2i-1)a_{n+2i-1}^{(r-1)} \, . \end{aligned}$$

### Lemma 9

59 $$\begin{aligned} a_{n}^{(r)}=\frac{1}{(r-1)!}\sum _{j=1}^{\infty }2^{2-2j}\frac{(j+r-2)!\Gamma (n+j+r-1)}{(j-1)!\Gamma (n+j)}\times (n+2j+r-2)a_{n+2j+r-2} \end{aligned}$$

### Proof

By using mathematical induction, at $``r=1''$: $a_{n}^{(1)}=\sum \nolimits _{j=1}^{\infty }2^{2-2j}(n+2j-1)a_{n+2j-1}$ (Eq. (58)). Assume that, the lemma holds for “r”. So, we have to show that:

60 $$\begin{aligned} a_{n}^{(r+1)}=\frac{1}{r!}\sum _{j=1}^{r+1}2^{2-2j}\frac{(j+r-1)! \Gamma (n+j+r)}{(j-1)!\Gamma (n+j)}(n+2j+r-1)a^{(r)}_{n+2j+r-1} \, . \end{aligned}$$

From Eq. (58) at $``r+1''$ and replacing *n* by $n+2i-1$:

61 $$\begin{aligned}&a_{n}^{(r+1)}=\sum _{i=1}^\infty 2^{2-2i} (n+2i-1)\frac{1}{(r-1)!}\sum _{j=1}^{\infty }2^{2-2j}\frac{(j+r-2)!}{(j-1)!}\!\times \frac{\Gamma (n\!+\!2i\!+\!j\!+\!r\!-\!2)}{\Gamma (n\!+\!2i\!+\!j\!-\!1)}\nonumber \\&\quad (n+2i+2j+r-3)a_{n+2i+2j+r-3} \end{aligned}$$

Let $$i + j - 1 = p$$. Then,

62 $$\begin{aligned}&a_{n}^{(r+1)}=\frac{1}{(r-1)!}\sum _{p=1}^{\infty }\sum _{\begin{array}{c} i,j=1,\\ i+j=p+1 \end{array}}^{p}2^{2-2p}\frac{(p-i+r-1)!}{(p-i)!}\times \frac{\Gamma (n+i+p+r-1)}{\Gamma (n+i+p)}\nonumber \\&\quad (n+2i-1)\times (n+2p+r-1)a_{n+2p+r-1} \end{aligned}$$

From Lemma 1 in Ref. Doha (1991):

63 $$\begin{aligned}&\sum _{i=1}^{p}(n+2i-1)\times \frac{(p-i+r-1)!\Gamma (n+i+p+r-1)}{(p-i)!\Gamma (n+i+p)}\nonumber \\ {}&\quad =\frac{(p+r-1)!\Gamma (n+p+r)}{q(p-1)!\Gamma (n+p)};\quad \forall r\ge 1. \end{aligned}$$

Then,

64 $$\begin{aligned} a_{n}^{(r+1)} =\frac{1}{r!}\sum _{p=1}^{\infty }2^{2-2p}\frac{(p+r-1)!\Gamma (n+p+r)}{(p-1)!\Gamma (n+p)}\times (n+2p+r-1)a_{n+2p+r-1} \, . \end{aligned}$$

The following theorem is the last needed step to set up the MC D-matrices.

### Theorem 10

The *r*th derivative of the MCPs is:

65 $$\begin{aligned} Q_{n}^{(r)}(x_{i})=\sum _{\begin{array}{c} k=0,\\ (n+k-r)\mathrm{{even}} \end{array}}^{n-r}b_{kn}^{(r)} Q_{k}(x_{i}) \, , \end{aligned}$$

where,

66 $$\begin{aligned} b_{kn}^{(r)}=\frac{1}{(r-1)!}2^{2k-2s}\frac{(s-k+r-1)!\Gamma (s+r)}{(s-k)!\Gamma (s+1)}n \, . \end{aligned}$$

### Proof

Substituting from Eq. (59) into Eq. (54) to get:

67 $$\begin{aligned}&f^{(r)}(x_{i})=\sum _{n=0}^{N}\frac{1}{(r-1)!}\sum _{j=1}^{\infty }2^{2-2j}\frac{(j+r-2)!\Gamma (n+j+r-1)}{(j-1)!\Gamma (n+j)}\times \nonumber \\&\quad (n+2j+r-2)a_{n+2j+r-2}Q_{n}(x_{i}) \, . \end{aligned}$$

Put $$l=n+2j+r-2$$ and $$2s=l+n-r$$:

68 $$\begin{aligned} f^{(r)}(x_{i})=\sum _{n=0}^{N}\frac{1}{(r-1)!}\sum _{\begin{array}{c} l=n-r,\\ (l+n-r)\mathrm{{even}} \end{array}}^{\infty }2^{2n-2s}\frac{(s-n+r-1)!}{(s-n)!}\times \frac{\Gamma (s+r)}{\Gamma (s+1)} la_{l} Q_{n}(x_{i}) \, . \end{aligned}$$

By differentiating Eq. (47) *r* times:

69 $$\begin{aligned} f^{(r)}(x_{i})=\sum _{l=r}^{N}a_{l} Q_{l}^{(r)}(x_{i} ) \, . \end{aligned}$$

Then, equating the coefficients of $a_{l}$ from Eqs. (68) and (69):

70 $$\begin{aligned} Q_{n}^{(r)}(x_{i})=\sum _{\begin{array}{c} k=0,\\ (k+n-r)\mathrm{{even}} \end{array}}^{n-r}\frac{1}{(r-1)!}2^{2k-2s}\frac{(s-k+r-1)!\Gamma (s+r)}{(s-k)!\Gamma (s+1)} nQ_{k}(x_{i}) \, , \end{aligned}$$

which proved the theorem.

Finally, the following corollary constructs the MC D-matrices. The construction will be easy due to the above lemmas, theorems, and steps.

### Corollary 11

Let *f*(*x*) be a differentiable function on the interval $[-1,1]$. Then,

71 $$\begin{aligned} f^{(r)}(x)=D^{(r)}[f(x)], \quad r=1, 2,..., N\, , \end{aligned}$$

where $D^{(r)}=[d_{ij}^{(r)}]; \quad i,j=0,1,...,N$ are square matrices of order $(N+1)$ and their entries are given by

72 $$\begin{aligned} d_{ij}^{(r)}=\sum _{n=r}^{N}\sum _{\begin{array}{c} k=0,\\ (k+n-r)\mathrm{{even}} \end{array}}^{n-r}\frac{2^{2n-1}}{N}c_{n}\theta _{j}\,b_{kn}^{(r)}Q_{n}(x_{j})\, Q_{k}(x_{i} ) \, . \end{aligned}$$

### Proof

By differentiating Eq.(47) *r* times w.r.t. *x*:

73 $$\begin{aligned} f^{(r)}(x)=\sum _{n=r}^{N}a_n Q_n^{(r)}(x) \, . \end{aligned}$$

Use Theorem (10) to get:

74 $$\begin{aligned} f^{(r)}(x_i)&=\sum _{n=r}^{N}\sum _{j=0}^{N} \frac{2^{2n-1}}{N}c_n Q_n(x_j)\theta _{j} \sum _{\begin{array}{c} k=0,\\ (k+n-r)\mathrm{{even}} \end{array}}^{n-r}b_{kn}^{(r)} Q_{k}(x_{i})f(x_j)\nonumber \\&=\sum _{j=0}^{N}\sum _{n=r}^{N} \sum _{\begin{array}{c} k=0,\\ (k+n-r)\mathrm{{even}} \end{array}}^{n-r} \frac{2^{2n-1}}{N}c_n Q_n(x_j)\theta _{j} b_{kn}^{(r)} Q_{k}(x_{i})f(x_j)\nonumber \\&=\sum _{j=0}^{N} d_{ij}^{(r)} f(x_j)\,, \end{aligned}$$

such that:

75 $$\begin{aligned} d_{ij}^{(r)}=\sum _{n=r}^{N}\sum _{\begin{array}{c} k=0,\\ (k+n-r)\mathrm{{even}} \end{array}}^{n-r}\frac{2^{2n-1}}{N}c_{n}\theta _{j}b_{kn}^{(r)}Q_{n}(x_{j}) Q_{k}(x_{i} ). \end{aligned}$$

Another form of the matrices can be obtained by using the trigonometric identity:

76 $$\begin{aligned} d_{ij}^{(r)}&=\sum _{n=r}^{N}\sum _{\begin{array}{c} k=0,\\ (k+n-r)\mathrm{{even}} \end{array}}^{n-r}\frac{2^{n-k+1}}{N} c_{n} \theta _{j} b_{kn}^{(r)} \cos \left( n \frac{j\pi }{N}\right) \cos \left( k\frac{i\pi }{N}\right) \, , \end{aligned}$$

and the periodic properties of the cosine function is:

77 $$\begin{aligned} d_{ij}^{(r)}=\sum _{n=r}^{N}\sum _{\begin{array}{c} k=0,\\ (k+n-r)\mathrm{{even}} \end{array}}^{n-r}\frac{2^{n-k+1}}{N}c_{n} \theta _{j} b_{kn}^{(r)}(-1)^{[\frac{nj}{N}]+[\frac{ki}{N}]}x_{nj-N[nj/N]}x_{ki-N[ki/N]} \, . \end{aligned}$$

## Error analysis and convergence

In the section, the error analysis and convergence discussions have been categorized into three subsections.

### Error upper-bound for D-matrices

This section is concerned with the roundoff error in the elements of the MC D-matrices. In finite precision arithmetic:

78 $$\begin{aligned} x_k^*=x_k+\delta \, \end{aligned}$$

where $\delta =\max _k{|\delta _k|}$, $\delta _k$ denotes a small error, with $|\delta _k|$ approximately equal to machine precision $\varepsilon$, and $x_k^*$ is the exact value while $x_k$ is the computed value with unit roundoff $\varepsilon = 2.22e-16$. The absolute errors of the quantities $x_kx_n$ are Baltensperger and Trummer (2003):

79 $$\begin{aligned} \left| x_k^*x_n^*-x_kx_n\right| =\delta _k+\delta _n-O\bigg (\frac{1}{N^2}\delta _k\bigg )-O\bigg (\frac{1}{N^2}\delta _n\bigg ) \, . \end{aligned}$$

Considering Eq. (77), the roundoff error on the matrix’s elements at $$ r=1$$ are given by:

80 $$\begin{aligned} d_{ij}^{(1*)}-d_{ij}^{(1)}&=\frac{4\theta _{j}}{N}\sum _{n=1}^{N}\sum _{\begin{array}{c} k=0,\\ (k+n-1)\mathrm{{even}} \end{array}}^{n-1}c_{n}n(-1)^{[nj/N]+[ki/N]} \Bigg ((\delta _{nj-N[nj/N]}+\delta _{ki-N[ki/N]})\nonumber \\&\quad -O\bigg (\frac{1}{N^{2}}\delta _{nj-N[nj/N]}\bigg ) -O\bigg (\frac{1}{N^{2}}\delta _{ki-N[ki/N]}\bigg )\Bigg )\nonumber \\&\le \frac{4\theta _{j}}{N}\left( \delta - O\left( \frac{1}{N^{2}}\delta \right) \right) \sum _{n=1}^{N} c_{n}n^{2}\nonumber \\&\le 4\theta _{j}\left( \delta - O\left( \frac{1}{N^{2}}\delta \right) \right) \left( \frac{N^{2}}{3}+\frac{1}{6}\right) \, . \end{aligned}$$

Hence, this order is in agreement with the order obtained in Ref. Elbarbary and El-Sayed Salah (2005).

For $r=2$, the error on the elements are given by:

81 $$\begin{aligned} d_{ij}^{(2*)}-d_{ij}^{(2)}&=\frac{2\theta _{j}}{N}\sum _{n=2}^{N}\sum _{\begin{array}{c} k=0, (k+n)\mathrm{{even}} \end{array}}^{n-2}c_{n} n(n^2-k^2)(-1)^{[nj/N]+[ki/N]} \Bigg (\delta _{nj-N[nj/N]}+\delta _{ki-N[ki/N]}\nonumber \\&\quad -O\bigg (\frac{1}{N^{2}}\delta _{nj-N[nj/N]}\bigg ) -O\bigg (\frac{1}{N^{2}}\delta _{ki-N[ki/N]}\bigg )\Bigg )\nonumber \\&\le \frac{2\theta _{j}}{N}\left( \delta - O\left( \frac{1}{N^{2}}\delta \right) \right) \sum _{n=2}^{N}c_{n}\left( \frac{2}{3}n^{4}+\frac{1}{2}n^{3}-\frac{13}{6}n^2+n\right) \nonumber \\&\le 2\theta _{j}\left( \delta - O\left( \frac{1}{N^{2}}\delta \right) \right) \left( \frac{2}{15}N^4+\frac{1}{8}N^3-\frac{1}{2}N^2+\frac{5}{8}N-\frac{23}{60}\right) \, . \end{aligned}$$

And so on, the roundoff error of the elements of the MC-matrices can be calculated for any order. In the end, the roundoff error of $d_{ij}^{(r)}$ observed to be $O(N^ {2r}\delta )$. The obtained roundoff error for the higher derivative is disturbing. But this issue does not affect due to the condition numbers.

### The condition numbers of MC D-matrices

It is known that the system is said to be ill-conditioned if its condition number is too large. Table 1 represents the condition numbers of MC D-matrices and Chebyshev D-matrices (Khalil et al. 2012) for different orders at different *N*. As shown, the condition number decreases by increasing the number of MC-GLQ points.

**Table 1:** Condition numbers for D-matrices

*N*	$D^1$		$D^2$		$D^3$		$D^4$	
	MCPs	CPs	MCPs	CPs	MCPs	CPs	MCPs	CPs
4	1.00e+00	1.56e+01	2.35e+02	1.51e+01	1.49e+07	7.62e+00	1.52e+08	1.30e+01
8	1.03e+00	8.07e+01	2.94e+00	1.87e+02	3.21e+00	4.61e+01	1.82e+02	4.07e+02
16	1.16e+00	1.37e+02	1.62e+00	8.57e+02	4.37e+00	3.36e+02	1.31e+01	5.23e+04
32	7.36e+00	3.96e+03	2.44e+01	8.21e+04	1.84e+02	6.37e+03	7.78e+01	1.25e+08
64	3.74e+01	2.36e+03	8.82e+02	4.22e+05	8.12e+03	1.31e+05	8.36e+04	3.25e+08

### Convergence analysis

In this section, we shall investigate and introduce some essential lemmas and theorems. These theorems and lemmas will be used to prove the boundedness and the convergence of the expansions.

#### Lemma 12

$$\left| Q_n(x)\right| \le 2^{1-n}$$, for $$n \ge 0$$.

#### Proof

In case of $n=0$, from Definition (8):

82 $$\begin{aligned} \left| Q_0(x)\right| =1<2. \end{aligned}$$

On the other hand, when $n>0$:

83 $$\begin{aligned} \left| Q_n(x)\right| =\left| 2^{1-n}\, T_n(x)\right| . \end{aligned}$$

So, by using the property (4):

84 $$\begin{aligned} \left| Q_n(x)\right| =\left| 2^{1-n}\, T_n(x)\right| \le \left| 2^{1-n}\,\right| =2^{1-n}\,. \end{aligned}$$

The above lemma shows that the roundoff error of approximation (47) tends to zero as *n* tends to infinity. The result neutralize the roundoff error of the elements of the high derivative MC D-matrices.

#### Theorem 13

Let $f(x)\in C^2[-1,1]$ can be approximated as in (47). Then,

- $$\left| a_n\right| \le \frac{A}{n^2}$$, for $$n > 1$$, where $$ A \ge \frac{M}{\pi }(2 + \pi )$$.
- E=$$\left| f(x)_{exact}- f(x)_{appr}\right| \le \frac{A}{2^N}$$  .

#### Proof

The technique of the proof as in Abd-Elhameed and Youssri (2014, 2019): from Eqs. (12) and (47):

85 $$\begin{aligned} a_n=\frac{1}{2^{1-2n} \pi } \int _{-1}^{1}\frac{f(x) Q_n(x)}{\sqrt{1-x^2}} \mathrm{{d}}x=\frac{1}{ \pi } \int _{0}^{\pi }f(\cos \theta ) \cos n \theta \, \mathrm{{d}}\theta \end{aligned}$$

Apply the integration by parts two times:

86 $$\begin{aligned} a_n=\frac{1}{n^2 \pi } \left[ (-1)^n f''((-1)^n)-f''(0) -\int _{0}^{\pi }f''(\cos \theta ) \cos n \theta \,\mathrm{{d}}\theta \right] \end{aligned}$$

Then,

87 $$\begin{aligned} \left| a_n\right| \le \frac{1}{n^2 \pi } \left[ M+M +M \pi \right] \le \frac{A}{n^2}. \end{aligned}$$

For the second item

88 $$\begin{aligned} E&=\left| \sum _{n=0}^{\infty }a_nQ_n(x) - \sum _{n=0}^{N}a_nQ_n(x)\right| = \left| \sum _{n=N+1}^{\infty }a_nQ_n(x)\right| \end{aligned}$$

89 $$\begin{aligned}&\le \left| \sum _{n=N+1}^{\infty }\frac{A}{n^2}2^{1-n}\right| \le A\left| \sum _{n=N+1}^{\infty }2^{1-n}\right| =A \left| \int _{N}^{\infty }2^{1-t} \mathrm{{d}}t\right| \le \frac{A}{2^N}. \end{aligned}$$

## Test functions

Hence the differentiation matrices have been constructed. We shall apply them to some test functions to show the efficiency of these matrices. Comparisons with exact solutions and other numerical methods have been made. The test will be started with a power function.

### Example 1

90 $$\begin{aligned} f(x)=x^8 \end{aligned}$$

Table 2, represents the maximum absolute error (MAE) of the fourth derivative at different values of *N*. Those results are compared by the method in Elbarbary and El-Sayed Salah (2005).

**Table 2:** The MAE for Example 1 at different values of *N*

*N*	8	12	14	16	32	64
MC D-matrices	7.3e$-$12	5.8e$-$10	7.3e$-$10	3.0e$-$09	4.1e$-$07	3.0e$-$03
Elbarbary and El-Sayed Salah (2005)	–	–	–	6.8e$-$09	4.5e$-$06	1.7e$-$03

The MAE is “7.3e$-$12” at $N=8$ for the presented method. While the MAE by the authors in Elbarbary and El-Sayed Salah (2005) is “6.8e$-$09” at $N=16$. This proved the efficiency and accuracy of this method.

### Example 2

91 $$\begin{aligned} f(x)=\sin x \end{aligned}$$

For a different values of *N*, the MAE for the fourth derivative of $f(x)=\sin x$ is shown in Table 3. Those results are compared by the method in Elbarbary and El-Sayed Salah (2005).

**Table 3:** The MAE for Example 2 at different values of *N*

*N*	14	16	32	64
MC D-matrices	8.7e$-$10	3.4e$-$09	8.1e$-$07	6.2e$-$04
Elbarbary and El-Sayed Salah (2005)	–	1.5e$-$08	5.1e$-$06	2.4e$-$03
Khalil et al. (2012)	1.1e$-$09	1.3e$-$09	–	–

The MC D-matrices have been tested as a derivative tool in the above section. But the essential task of the MC D-matrices is solving ODEs and real-life applications represented by BVPs.

## Proposed method

The technique of the D-matrix is effortless to apply. Consider the ODE of order *r* as follow:

92 $$\begin{aligned} y^{(r)}(x)=e(x) h(y)+s(x); \quad -1\le x\le 1 \, , \end{aligned}$$

with appreciate and suitable initial and boundary conditions as regular, where *e*(*x*), *s*(*x*) are real functions of *x* and *h*(*y*) linear or nonlinear function of *y*.

From Eq. (74):

93 $$\begin{aligned} y^{(r)}(x_i)=\sum _{j=0}^{N} d_{ij}^{(r)} y(x_j) \, . \end{aligned}$$

Substitute into the ODE (Eq. (92)):

94 $$\begin{aligned} \sum _{j=0}^{N} d_{ij}^{(r)} y(x_j)=e(x_i) h(y(x_i))+s(x_i); \quad i=0,1,\dots ,N. \end{aligned}$$

A similar procedure will be done with the initial and boundary conditions. The equations mentioned above, (94), with those from the initial and boundary conditions form a system of algebraic equations of $N+1$ unknowns at maximum ($y(x_i)$, i=0,1,...,N). The number of unknowns depends on the given conditions. The algebraic system will be solved by any solver analytically or numerically. Algorithm (1) has been created to enable the readers to code a program easily.
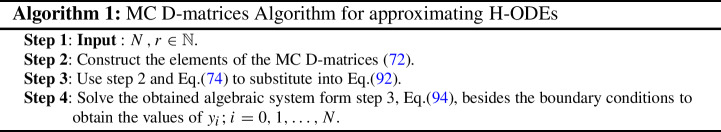


## Numerical examples

In this section, we apply the MC D-matrices to some H-ODEs. Then, comparisons with exact solutions, other numerical methods, and the bvp5c MATLAB function (if possible) have been made. But the H-ODEs must be transformed into a system of 1st order ODE to use bvp5c. This transformation will reduce the efficiency of the bvp5c due to the magnification of the variables. The parameters of bvp5c were taken as RelTol = 1e$-$16 and AbsTol = 1e$-$16. That codes of the MATLAB software run using i7-4500 CPU @ 1.80GHz Intel, that supported by SSD hard disk.

### Example 3

Consider the following nonlinear fourth-order BVPs:

95 $$\begin{aligned} 16y^{(4)}(x)+\frac{(x+1)^2}{4(1+y^2(x))}= & {} -72\left( 1-\frac{5}{2}(1+x)+\frac{5}{4}(1+x)^2 \right) \nonumber \\&+\frac{0.25(x+1)^2}{1+\left( 0.5(1+x)-0.25(1+x)^2 \right) ^6}; -1\le x\le 1. \end{aligned}$$

subject to: $y(-1)= y(1)=y^{(1)}(-1)=y^{(1)}(1)=0.$ The exact solution: $y=\frac{1}{64}(1-x^2)^3$ ,

**Fig. 1:**
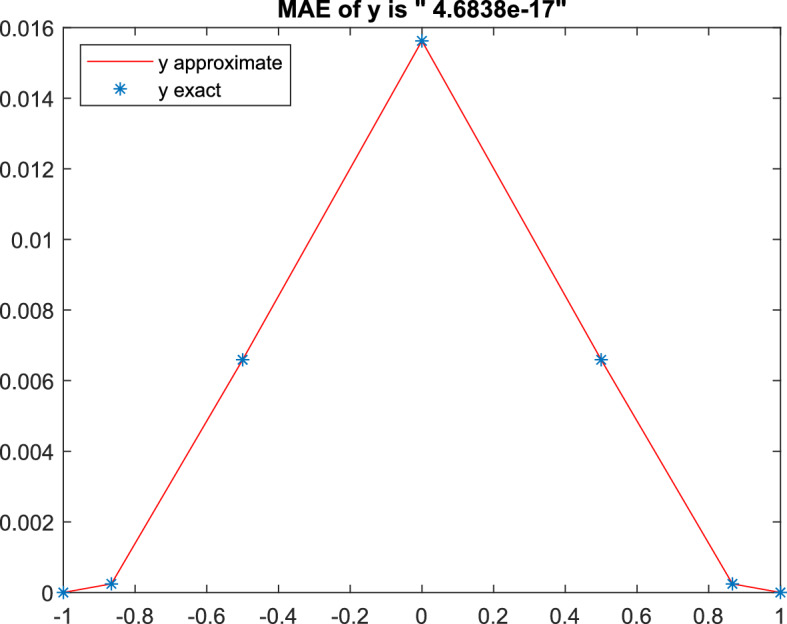
Approximated and exact solution of Example 3 at $N=6$

Let $y(x)=\sum \nolimits _{n=0}^{6}a_{n}Q_{n}(x)$. Thus, the elements of the MC D-matrices, Eq. (72), are:

96 $$\begin{aligned} d_{ij}^{(4)}=\sum _{n=r}^{6}\sum _{\begin{array}{c} k=0,\\ (k+n-r)\mathrm{{even}} \end{array}}^{n-r}\frac{2^{2n-1}}{6}c_{n}\theta _{j}\,b_{kn}^{(r)}Q_{n}(x_{j})\, Q_{k}(x_{i} ) \, , \end{aligned}$$

such that: $r=1,4$, $x_{i}=\cos \frac{\pi i}{N}$, and $i,j=0,1,\ldots ,6$,

where $c_n$, $\theta _j$, and $b_{kn}^{(r)}$ are defined as in Eqs. (49), and (66), respectively.

Use Eq. (74) and substitute into Eq. (95):

97 $$\begin{aligned} \begin{aligned} 16\sum _{j=0}^{6} d_{ij}^{(4)} y(x_j)+\frac{\left( x_i+1\right) ^2}{4\left( 1+y^2(x_i)\right) }&=-72\left( 1-\frac{5}{2}(1+x_i)+\frac{5}{4}(1+x_i)^2 \right) \\&\quad +\frac{0.25(x_i+1)^2}{1+\left( 0.5(1+x_i)-0.25(1+x_i)^2 \right) ^6}\, \quad i=0,1,\ldots ,6. \end{aligned} \end{aligned}$$

The initial/boundary conditions will be:

98 $$\begin{aligned} \begin{array}{lcl} y(-1)=0&{}:&{} y(x_0)=0\,, \\ y(1)=0&{}:&{} y(x_6)=0\,, \\ y^{(1)}(-1)=0&{}:&{} \sum \limits _{j=0}^{6} d_{0j}^{(1)} y(x_j)=0\,, \\ y^{(1)}(1)=0&{}:&{} \sum \limits _{j=0}^{6} d_{6j}^{(1)} y(x_j)=0\,. \end{array} \end{aligned}$$

Use any solver to solve the system (97), (98) to get the values of $y(x_i)$; $i=0,1,\ldots ,6$.

MC D-matrices got MAE “4.7e$-$17” (double-precision) at $N=6$. In Khalil et al. (2012), the authors got “1.1e$-$14” but at $N=10$. While in Lu et al. (2019), the MAE is only “1.1e$-$07” using 11 points. On the other hand, the bvp5c Matlab function got “5.5e$-$12” using 7 points. But to get “1.2e$-$17”, 487 points must be used. That means the presented method is more accurate and efficient. Figure 1, represented the approximated and the exact solution.

### Example 4

Consider the linear eighth-order BVP:

99 $$\begin{aligned} 256y^{(8)}+\frac{1}{2}(1+x)y=\left( -48-\frac{15}{2}(1+x)-\frac{1}{8}(1+x)^3\right) e^{\frac{1}{2}(1+x)}; \quad -1\le x\le 1\,, \end{aligned}$$

subject to:

100 $$\begin{aligned} \begin{array}{c} y(-1)=y(1)=0,\quad y^{(1)}(-1)=0.5,\quad y^{(1)}(1)=-0.5e,\\ y^{(2)}(-1)=0,\quad y^{(2)}(1)=-e, \quad y^{(3)}(-1)=-0.375, \quad y^{(3)}(1)=-1.125e\, , \end{array} \end{aligned}$$

and exact solution is

101 $$\begin{aligned} y(x)=\frac{1}{4}(1-x^2)e^{0.5(1+x)}. \end{aligned}$$

In that example, H-BVP (Eq. (99)) has been solved directly and by transforming it into a system of lower-order (4th, 2nd and 1st) ODEs, respectively.

**Table 4:** The point wise AE for Example 4

*x	MC D-matrices							bvp5c	(Ogunrinde and Ojo 2018)
	Direct	System							
		4th	2nd		1st				
	$N=11$	$N=10$	$N=5$	$N=10$	$N=4$	$N=5$	$N=10$	$N=10$	$N=10$
$-$1	1.7e$-$16	9.5e$-$18	0	2.9e$-$17	1.4e$-$17	9.6e$-$18	1.2e$-$17	0	0
$-$0.8	2.7e$-$03	3.7e$-$05	7.8e$-$04	8.9e$-$12	2.1e$-$03	1.1e$-$04	2.7e$-$13	2.9e$-$07	1.2e$-$05
$-$0.6	4.9e$-$03	3.7e$-$04	1.6e$-$03	6.9e$-$13	5.7e$-$03	2.0e$-$04	3.6e$-$13	2.9e$-$06	1.5e$-$04
$-$0.4	5.7e$-$03	1.1e$-$03	2.2e$-$03	2.7e$-$12	8.5e$-$03	1.2e$-$04	6.1e$-$12	8.8e$-$06	5.5e$-$04
$-$0.2	4.9e$-$03	1.9e$-$03	2.6e$-$03	1.7e$-$11	9.3e$-$03	1.0e$-$04	4.4e$-$12	1.5e$-$05	1.3e$-$03
0	2.7e$-$03	2.3e$-$03	2.5e$-$03	2.3e$-$13	8.1e$-$03	3.1e$-$04	1.3e$-$12	1.8e$-$05	2.2e$-$03
0.2	2.9e$-$04	2.0e$-$03	2.1e$-$03	1.8e$-$11	5.5e$-$03	4.0e$-$04	6.3e$-$12	1.6e$-$05	2.8e$-$02
0.4	1.5e$-$03	1.2e$-$03	1.4e$-$03	2.6e$-$12	2.5e$-$03	3.4e$-$04	2.3e$-$12	9.3e$-$06	3.8e$-$03
0.6	2.0e$-$03	4.0e$-$04	6.8e$-$04	7.8e$-$14	1.5e$-$04	1.8e$-$04	1.9e$-$12	3.2e$-$06	4.2e$-$03
0.8	1.3e$-$03	4.1e$-$05	1.9e$-$04	9.3e$-$12	6.6e$-$04	4.2e$-$05	1.7e$-$12	3.2e$-$07	4.3e$-$03
1	2.5e$-$17	9.6e$-$17	1.4e$-$17	3.3e$-$17	4.2e$-$17	3.5e$-$17	8.5e$-$17	0	4.4e$-$03

Table 4, represents the point wise absolute errors (AEs) for Example 4 in a comparison with those in Ogunrinde and Ojo (2018) and the bvp5c Matlab function. This comparison showed the privilege and the demonstration of the high accuracy and the the efficiency of MC D-matrices

The following two examples discuss two real-life applications.

### Example 5

This example treats with general unified Magnetohydrodynamics (MHD) boundary-layer flow of a viscous fluid (Karkeraa et al. 2020). The authors transformed the boundary-layer equations into a governing problem over an unbounded domain. This governing problem takes the form of Falkner–Skan-type equation:

102 $$\begin{aligned} y^{(3)}+yy^{(2)}+\beta \left( \epsilon ^2-\left( y^{(1)}\right) ^2 \right) +M^2\left( \epsilon -y^{(1)}\right) =0; \quad 0\le x < \infty \,, \end{aligned}$$

subject to:

103 $$\begin{aligned} y(0)= 0, \quad y^{(1)}(0)=1-\epsilon , \quad y^{(1)}(\infty )=\epsilon . \end{aligned}$$

where:

$\epsilon$ : the parameter of composite velocity.

$\beta$ : the moving boundary rate.

*M* : Hartmann number.

For more details, refer to Karkeraa et al. (2020). The authors of Karkeraa et al. (2020) discussed several cases. Here, we chose one of them as a sample, for $\epsilon =M=0$ and $\beta =-1$ with the exact solution $y=\sqrt{2}\tanh \left( \frac{x}{\sqrt{2}}\right)$. After the transformation $\eta =1-2e^{-x}$ , the MAE reaches $10^{-4}$ at $N=38$ using the MC D-matrices. While the MAE almost tended to $10^{-4}$ after the 6th level of resolution in Karkeraa et al. (2020). The 6th level of resolution in Karkeraa et al. (2020) means $2^{6+1}-1=127$ unknowns or iterations. This proved the dominance of the accuracy. Also, as a privilege of the presented method, the MC D-matrices are very easy to apply than the techniques that used in Karkeraa et al. (2020), Haar wavelet collocation and Haar wavelet quasilinearization. In the process of the transformation of the domain, the condition $y^{(1)}(\infty )=\epsilon$ has been lost. Consequently, the bvp5c Matlab function cant be used due to the insufficient number of conditions. However, our method can be applied without any problems.

In the introduction, the importance of BVPs was mentioned. This importance has appeared in our lives as in the pandemic of COVID-19.

### Example 6

The authors in Rong et al. (2020) and Moore and Okyere (2022) discussed the spread of COVID-19. They admitted that the rapid spread was due to diagnosis delay and lack of resources. The model of transmission of COVID-19 was investigated in Rong et al. (2020). The following model is modified in Moore and Okyere (2022):

104 $$\begin{aligned} \begin{array}{l} y'_1=-(1-\alpha )\left( b_1 y_3+b_2 y_4+b_3 y_5+b_4 y_8\right) y_1-q_1 y_1+q_2 y_2 \, , \\ y'_2=q_1 y_1-q_2 y_2\\ y'_3=(1-\alpha )\left( b_1 y_3+b_2 y_4+b_3 y_5+b_4 y_8\right) y_1-\omega y_3 \, ,\\ y'_4=\phi \omega y_3 -\left( \beta + \mu \right) y_4 \, , \\ y'_5=\left( 1-\phi \right) \omega y_3 -\left( \gamma + \mu \right) y_5 \, ,\\ y'_6=\beta y_4 +\gamma y_5 -\left( m+ \mu \right) y_6 \, ,\\ y'_7=m y_6 \, ,\\ y'_8=f_1 y_3 +f_2 y_4 + f_3 y_5 -\left( d+ \delta \right) y_8 \, . \end{array} \end{aligned}$$

For the initial conditions, all numeric parameters, and the meaning of the variables and the parameters, refer to Rong et al. (2020), Table 3. Moore and Okyere (2022) presented four strategies to handle the above system and described the model for 100 days as a time interval. They used the fourth-order Runge–Kutta forward–backward sweep method. To reach 100 days, they ought to iterate the equation a considerable number of iterations. As a sample, the first case has been examined by our method. To understand the presented graph, some notions will be explained in Table 5.

**Table 5:** COVID-19 model parameters description (Example 6)

Parameter	Description
$\alpha$	The personal protection
$\beta$	The early diagnosis treatment
$\gamma$	The delay diagnosis treatment
$\delta$	The environment spraying
*x*	The time in days
$\phi$	The proportion of the infectious with a timely diagnosis
$y_3$	The exposed members of the population $y_1$

**Fig. 2:**
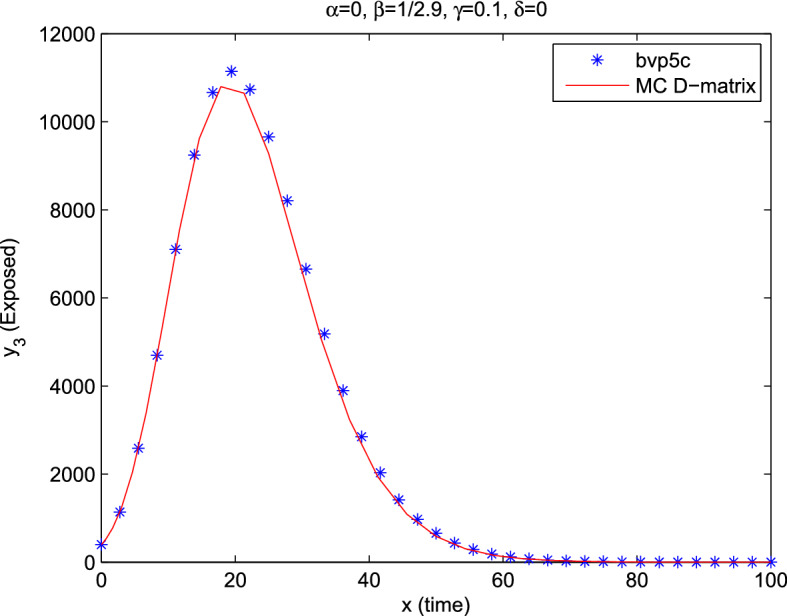
Exposed population for Example 6 of COVID-19

By changing the range of the dependent variable (time) from [0, 100] to $[-1,1]$, Fig. 2 represents the exposed population ($y_3$) during a hundred days approximated by the MC D-matrices and the bvp5c Matlab function. The figure is identical to the same case in Moore and Okyere (2022) with a few numbers of iterations. Also, the Matlab function reported that the maximum error of bvp5c is 1.270e+04. This showed that our procedure is more efficient, and it is effortless to apply to the system than the method in Moore and Okyere (2022).

Finally, the following example will discuss a famous elastic foundation problem.

### Example 7

Consider the 4th ODE for the ill-posed problem beam (Agarwal et al. 2020; Hussain et al. 2016; Dong et al. 2014):

105 $$\begin{aligned} y^{(4)}=1-y; \quad 0\le x \le 1\,, \end{aligned}$$

subject to:

106 $$\begin{aligned} y(0)= 0, \quad y^{(1)}(0)=0, \quad y^{(2)}(0)=0, \quad y^{(3)}(0)=0, \end{aligned}$$

with exact solution:

107 $$\begin{aligned} y=1-\frac{\left( e^{\sqrt{2}x}+1\right) e^{-x/\sqrt{2}} \cos \left( \frac{x}{\sqrt{2}} \right) }{2} \end{aligned}$$

where, *y* represent the bar deviation.

**Fig. 3:**
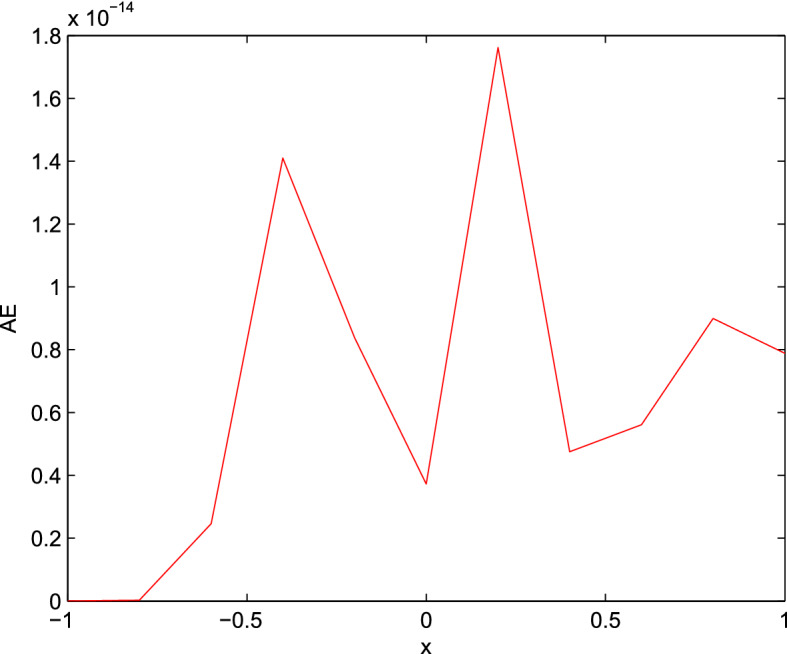
AE for Example 7 at $N = 10$ using system of 1st order differential equations

By applying the same routine, we get the results that have been shown in Table 6. These results demonstrate the accuracy and the efficiency over the bvp5c. Also, the MC D-matrices obtained more accurate results than the results in Agarwal et al. (2020). Figure 3 represents the AE using the system of $1^{st}$ order differential equations at $N=10$.

**Table 6:** The point wise AE for Example 7 using 11 points

*x*	MC D-matrices		bvp5c
	Direct	System of 1st	
$-$1	1.3e$-$18	1.7e$-$18	0
$-$0.8	1.8e$-$09	2.8e$-$17	3.1e$-$06
$-$0.6	1.3e$-$08	2.5e$-$15	5.0e$-$05
$-$0.4	4.4e$-$08	1.4e$-$14	2.5e$-$04
$-$0.2	1.0e$-$07	8.5e$-$15	8.0e$-$04
0	2.0e$-$07	3.7e$-$15	2.0e$-$03
0.2	3.4e$-$07	1.7e$-$14	4.0e$-$03
0.4	5.5e$-$07	4.8e$-$15	7.5e$-$03
0.6	8.2e$-$07	5.6e$-$15	1.3e$-$02
0.8	1.2e$-$06	9.0e$-$15	2.0e$-$02
1	1.6e$-$06	7.9e$-$15	3.1e$-$02

Finally, we will proceed to the last example that describes the chaotic velocity nature of turbulent flows.

### Example 8

Consider the 7th ODE (Akram and Beck 2015):

108 $$\begin{aligned} y^{(7)}=y-35e^x-14xe^x; \quad 0\le x \le 1 \, , \end{aligned}$$

subject to:

109 $$\begin{aligned} y(0)= & {} 0, \quad y^{(1)}(0)=1 \quad y^{(2)}(0)=0, \quad y^{(3)}(0)=-3, \quad y^{(4)}(0)=-8,\nonumber \\ y^{(5)}(0)= & {} -15, \quad y^{(6)}(0)=-24, \end{aligned}$$

with exact solution: $y=x(1-x)e^x$, where, *y* is particles’ velocity for a limited time.

**Table 7:** The MAE for Example 8

*N*	MC D-matrices		bvp5c	(Akram and Beck 2015)
	Direct	System of 1st		
3	–	1.34e$-$01	1.82e$-$06	–
10	1.77e$-$03	3.24e$-$12	1.32e$-$09	1.82e$-$01
12	4.99e$-$09	1.95e$-$14	4.41e$-$10	2.33e$-$08
15	3.82e$-$07	6.53e$-$14	1.15e$-$10	1.67e$-$08

The following results can be concluded from Table 7:

- MC D-matrices are more efficient and accurate than the method used in Akram and Beck (2015) as a direct method without transformation.
- By transforming the given problem into seven 1st order ODEs, MC D-matrices are still more efficient and accurate than the bvp5c Matlab function.
- Since the bvp5c Matlab function deals with the 1st order ODEs only so, in our case, the bvp5c Matlab function handles $5(N+1)$ variables. On the other hand, the direct method in MC D-matrices runs $N+1$ only. That means high efficiency.

## Conclusion

Some basic properties and concepts for the MCPs have been introduced. These concepts are used to set up higher-order MC D-Matrices. Then, we investigated the error analysis for the proposed method and D-matrices. This analysis included three items. The first item was the upper roundoff error for the elements of MC D-matrices for those matrices. While the second item was the condition number of MC D-matrices. Finally, the convergence of the approximation and the truncation error was presented. Consequently, the MC D-matrices has been tested by two different test functions. To prove the efficiency and the power of that technique, we apply it to various examples. Those examples represented six different categories. The first example was nonlinear H-BVP. In contrast, the second introduced a linear H-BVP. The other four examples deal with real-life applications. One about the MHD and the results were very efficient. Eight nonlinear first-order ODEs representing a model of COVID-19 were solved in the second example. The third example is about the ill-posed problem beam. Finally, the last application was about the chaotic velocity of the particles in the turbulent flows. Comparisons with other methods and bvp5c have been made if applicable. Due to insufficient conditions, the bvp5c Matlab function failed to solve the MHD example. In contrast, the represented method has no problems. Generally, the technique of MC D-matrices is reliable and easy to apply. Almost one algorithm may be used to solve most problems, whether linear ODE, nonlinear ODE, or system of ODEs. The MC D-Matrices can be extended to deal with partial differential equations in future work. Moreover, it can be generalized and applied to fractional calculus.
